# Post-Harvest Quality Changes and Molecular Responses of Epidermal Wax in ‘Munage’ Grapes with *Botrytis cinerea* Infection

**DOI:** 10.3390/ijms26083468

**Published:** 2025-04-08

**Authors:** Yu Wang, Yunhao Lv, Tong Han, Yidong Liu, Ying Jiang

**Affiliations:** 1 College of Smart Agriculture (Research Institute), Xinjiang University, Urumqi 830046, China; wangyu2023@xju.edu.cn; 2 School of Food Science and Technology, Shihezi University, Shihezi 832003, China; b20223060525@cau.edu.cn (Y.L.); hantong147762@163.com (T.H.)

**Keywords:** ‘Munage’ grapes, *Botrytis cinerea*, epicuticular wax, wax composition, storage

## Abstract

This study aimed to investigate the impact of Botrytis cinerea (*B. cinerea*) on the post-harvest quality of ‘Munage’ grapes and their molecular mechanism. The results showed that *B. cinerea* significantly reduced the post-harvest quality of ‘Munage’ grapes, which was manifested by an increase in incidence and rot rate, a significant increase in weight loss rate and fruit color difference, and a significant decrease in fruit firmness. In addition, *B. cinerea* infection significantly changed the reactive oxygen species and antioxidant enzyme activities of ‘Munage’ grapes, including increasing the H_2_O_2_ content and O_2_^−^ generation rate as well as changing the superoxide dismutase (SOD), glutathione (GSH), catalase (CAT), and peroxidase (POD) activities. *B. cinerea* also significantly changed the wax structure and content of ‘Munage’ grapes, causing the wax to completely dissolve and disappear and reducing the relative content of wax components. Through RNA-seq analysis, it was found that after *B. cinerea* infection, 49 differentially expressed genes (DEGs) related to fatty acid synthesis, extension, cutin and wax synthesis, and wax transport showed up-regulation or down-regulation, and 12 different transcription factors (TFs) also showed significant differential expression. These TFs were correlated with DEGs related to wax synthesis and metabolism, indicating that they may play an important role in the epidermal wax changes in ‘Munage’ grapes caused by *B. cinerea*. This study revealed the impact of *B. cinerea* on the post-harvest quality of ‘Munage’ grapes and their molecular mechanism and provided a scientific basis for grape disease prevention and quality maintenance.

## 1. Introduction

The ‘Munage’ grape, a native variety of the Xinjiang Autonomous Region in China, is widely grown due to its adaptability and strong stress resistance [1]. In recent years, ‘Munage’ grape has become popular with consumers on account of its sweet flavor and large fruit size. However, being a non-climacteric fruit, table grapes do not undergo further ripening after harvest. During post-harvest storage, table grapes possess thin skin and a relatively low pH level, which renders them susceptible to fungi and pathogenic bacteria [2]. This vulnerability can result in the decline of table grapes’ quality, such as water loss, browning, rotting, and deterioration [2]. One of the drivers of fungal infection of grapes is’gray mold’, which is mainly caused by *Botrytis cinerea* (*B. cinerea*), a facultative parasitic fungus belonging to one of the most geographically widespread groups of plant pathogens [1,2]. *B. cinerea*-induced postharvest physiological disorders, particularly those mediated through host resistance modulation, have been extensively reported [3,4]. For example, postharvest kiwifruit is highly susceptible to *B. cinerea*, leading to elevated disease incidence and lesion expansion. Notably, exogenous indole-3-acetic acid (IAA) treatment effectively alleviates postharvest physiological disorders in kiwifruit, involving enhanced defense enzyme activities and metabolic reprogramming, particularly activation of phenylpropanoid, terpenoid, and carbohydrate metabolism pathways, as revealed by a metabolomic analysis [3]. Zhu et al. discovered that the ethylene response transcription factor VaERF16 played a crucial role in grapes’ immune response against *B. cinerea*. Moreover, they revealed that the VaERF16-VaMYB306 genetic module positively regulated grapevine resistance to *B. cinerea* by modulating the JA/ET signaling pathway [4]. Li et al. determined that *B. cinerea* upregulated VvPG, which played a major role in cell wall degradation associated with the spike stalk browning of ‘Munage’ grapes [5]. Therefore, understanding how *B. cinerea* affects the post-harvest physiology of ‘Munage’ grape is essential for improving its quality and economic value.

Wax, a robust hydrophobic layer covering the fruit surface, not only maintains fruit freshness but also resists cold damage and pests, thereby extending the fruit’s shelf life [6]. Fruit cuticular wax is grouped into two types: the crystallized “epicuticular wax” and the “intracuticular wax” that is attached to the underlying epidermal cell walls [7]. The epicuticular wax is composed of very-long-chain fatty acids (VLCFAs) and their derivatives; however, plant epidermal cells produce a variety of secondary metabolites, including fatty acids, aldehydes, esters, alkanes and alcohols as well as cyclic compounds like triterpenoids and sterols [6,8]. “Korla” pear epicuticular wax presented with plate-like crystals and was mainly composed of olefins, fatty acids, and alkanes [9]. Yang et al. investigated the wax of four different typical grape varieties and found that grapes’ epicuticular wax had four kinds of irregular flaky crystal morphologies; regarding its wax content distribution, terpenoids were mainly found in the intracuticular wax, fatty acids in the epicuticular wax, and hydrocarbons uniformly in all wax [10].

Mounting evidence has indicated that epicuticular wax’s structure and components might be changed after fungi and pathogenic bacterial infection [11,12]. For example, the fungus *Colletotrichum gloeosporioides* infecting the tomato epicuticular wax was accompanied by stage-specific transcription, which was reflected in the significant up-regulation of tomato defense gene response [13]. *B. cinerea* infection is one major type of fungi infection during fruit post-harvest storage, in which infecting *B. cinerea* produces some hydrolytic enzymes that directly degrade the epicuticular wax and affect plant hormone networks [14,15]. A preliminary study showed that the infection of *B. cinerea* induced the enzyme activity of the defense system in blueberry, degraded the content of epidermal wax around the mycelium, and delayed the down-regulated expression of wax-related genes [16]. Additionally, the effects of epidermal wax compounds in table grapes on the growth, germination, and gene expression of *B. cinerea* has been demonstrated [17]. However, the specific infection mechanism of *B. cinerea* on ‘Munage’ grapes is still unclear, and there are few reports on the possible mechanism of *B. cinerea*-infected epicuticular wax composition and the expression of genes related to epidermal wax transport in ‘Munage’ grapes.

Therefore, the present study aimed to probe the impacts of *B. cinerea* inoculation on the composition and transcription factors of epidermal wax in ‘Munage’ grapes. The content of grapes’ epidermal wax was determined by gas chromatography-mass spectrometry (GC-MS), and a further analysis of the gene expression levels of ‘Munage’ grapes’ epidermal wax was carried out to investigate the response following *B. cinerea* inoculation. Moreover, the underlying mechanism of epicuticular wax composition infected by *B. cinerea* and the genes related to epidermal wax transport were further explored.

## 2. Results

### 2.1. B. cinerea Infection Decreased the Postharvest Quality in ‘Munage’ Grapes

**Figure 1:**
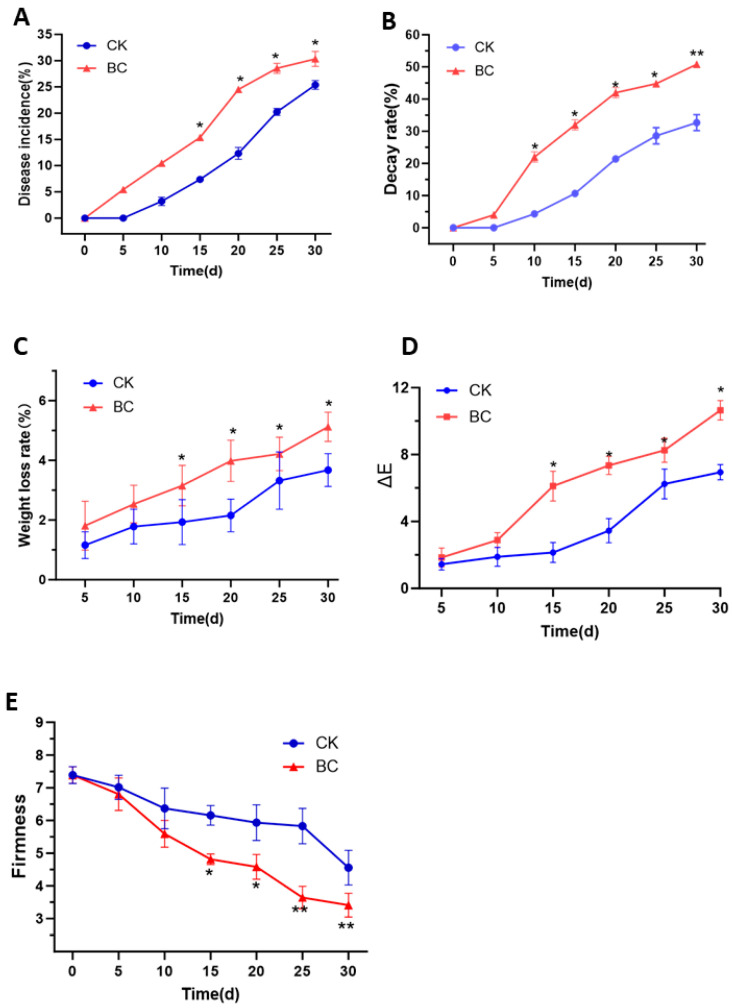
Effects of *B. cinerea* on (**A**) disease incidence, (**B**) decay rate, (**C**) weight loss rate, (**D**) rot rate, and (**E**) firmness in ‘Munage’ grapes. * Denotes significance at *p* < 0.05; ** denotes significance at *p* < 0.01.

The changes in disease incidence (Figure 1A) and decay rate (Figure 1B) exhibited progressive increases during storage time. *B. cinerea* inoculation induced significantly higher disease incidence (*p* < 0.05) and accelerated rot progression (*p* < 0.01) compared to the CK group. Weight loss (Figure 1C) and fruit color difference (Figure 1D) increased in all samples during storage. Notably, the weight loss rate and fruit color difference in the BC group were higher than those in the CK group (*p* < 0.05). Firmness decreased over time in both groups (Figure 1E). However, *B. cinerea* infection caused a marked reduction in firmness from day 15 onward (*p* < 0.05).

### 2.2. B. cinerea Infection Changed Active Oxygen and Antioxidant Enzyme Activities of ‘Munage’ Grapes

**Figure 2:**
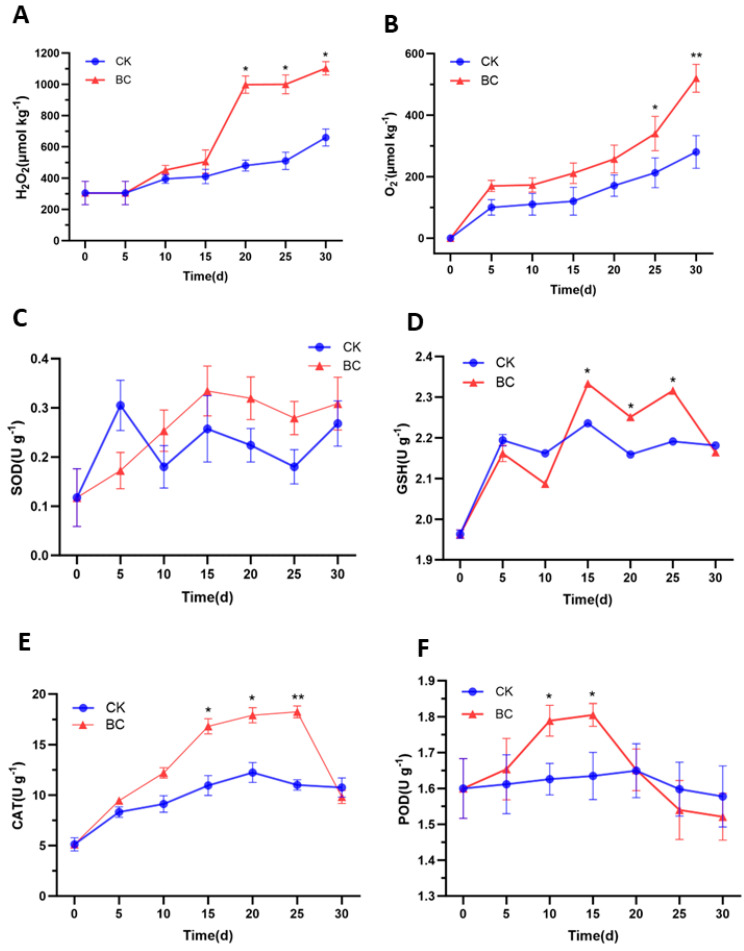
The active oxygen and antioxidant enzyme activities of ‘Munage’ grapes. (**A**) The content of H_2_O_2_; (**B**) production rate of O_2_^−^; and (**C**–**F**) relative content of SOD, GSH, CAT, and POD. * and ** denote significance at *p* < 0.05, *p* < 0.01.

H_2_O_2_ content and O_2_^−^ production rate exhibited synchronous trends (Figure 2A,B), and H_2_O_2_ and O_2_^−^ levels in the BC group were significantly higher than those in the CK group after 20 and 25 days of storage (*p* < 0.05). The superoxide dismutase (SOD) and glutathione (GSH) activities first increased and then declined (Figure 2C,D). In the BC group, SOD activity increased from day 10 onward, though no significant difference among CK was observed. In contrast, GSH activity in the BC group surged significantly at days 15, 20, and 25 (*p* < 0.05) before dropping to CK levels at 30 d. Figure 2E showed that the catalase (CAT) activity in the BC group demonstrated a sustained upward trend, peaking at 15 and 25 d post-inoculation with significant elevation compared to the CK group (*p* < 0.05) before declining slightly. In Figure 2F, peroxidase (POD) activity in the BC group significantly decreased at 10 and 15 d (*p <* 0.05), while the CK group maintained stability.

### 2.3. B. cinerea Infection Changed the Wax Structure and Amount in ‘Munage’ Grapes

**Figure 3:**
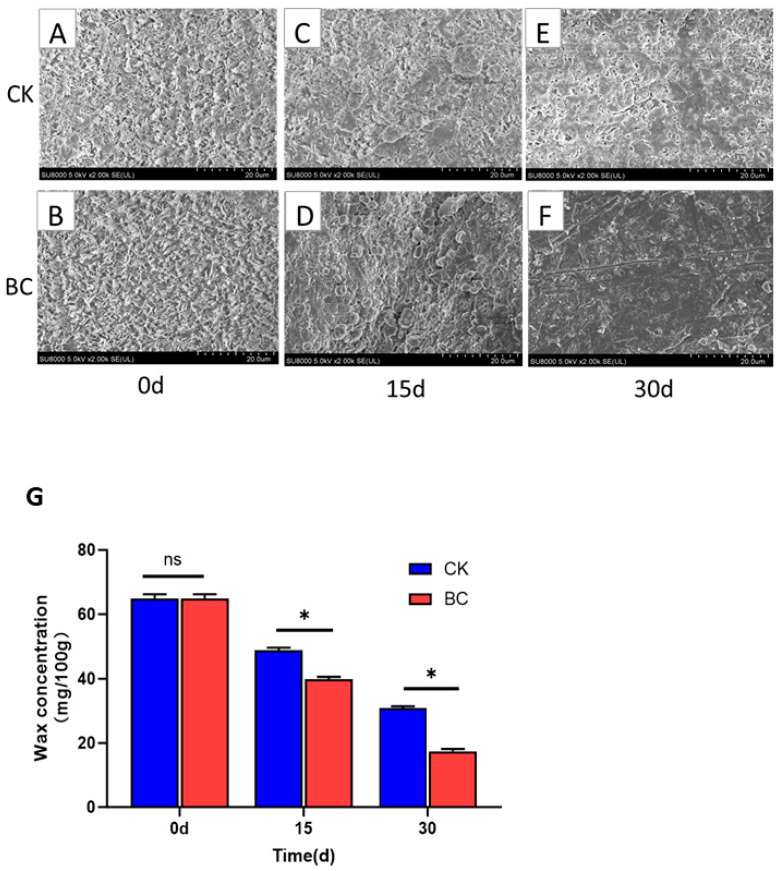
Effects of *B. cinerea* on waxy structure and amount in ‘Munage’ grapes. (**A**–**F**) Waxy structure of CK and BC groups was observed using SEM (×1000 magnification); (**G**) changes in wax amount after infection with *B. cinerea*. * denotes significance at *p* < 0.05, ns denotes no significant difference.

At 0 d, the epidermal wax of ‘Munage’ grapes showed a single shape and was stratified by many tubules (Figure 3A,B). After 15 days of storage, although the wax in the control group grapes was still flaky, its content had decreased (Figure 3C). However, in the *B. cinerea*-inoculated group, the cuticular wax of grapes began to dissolve, and fungal conidia were observed dispersed around the areas of wax degradation (Figure 3D). After 30 days of storage, it was observed that the stacked flake wax appeared to dissolve off the grapes in the control group, and the collapsed wax was continuously reduced, while the wax dissolved and disappeared from *B. cinerea*-infected grapes (Figure 3E,F). This result was further confirmed by the effect of *B. cinerea* on the wax amount of ‘Munage’ grapes; during the storage periods of 15 d and 30 d after inoculating, *B. cinerea* decreased the amount of wax by 18.47% and 43.54% when compared to the grapes in control group (Figure 3G, *p* < 0.05).

### 2.4. B. cinerea Infection Decreased the Wax Components in ‘Munage’ Grapes

**Figure 4:**
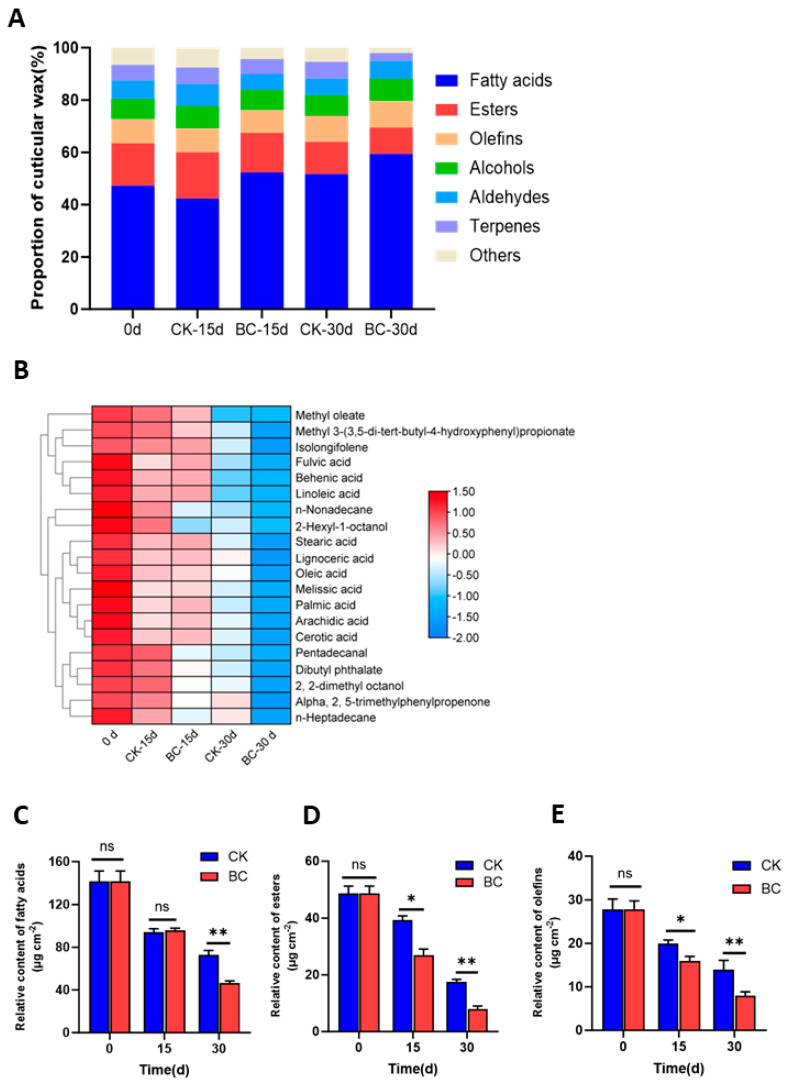
Effects of *B. cinerea* on waxy components in ‘Munage’ grapes. (**A**) The contents of waxy components. (**B**) Heatmap of waxy components in CK and BC groups at 0, 15, and 30 d. (**C**–**E**) The relative content of fatty acids, esters, and olefins. * and ** denote significance at *p* < 0.05, *p* < 0.01, ns denotes no significant difference.

We next assessed whether *B. cinerea* affected the epicuticular epidermal wax components in ‘Munage’ grapes. The epicuticular wax of ‘Munage’ grapes consists of fatty acids, olefins, alcohols, esters, aldehydes, terpenes, and other components. Among them, fatty acids were the most significant component, accounting for 47.26% of the total epidermal wax, followed by esters and olefins, accounting for 16.23% and 9.28% (Figure 4A). After infection with *B. cinerea* for 15 and 30 d, the proportions of epidermal wax changed in comparison with the CK group, while the seven kinds of wax components in ‘Munage’ grapes did not change. There were 52 compounds identified in ‘Munage’ grapes’ wax (Supplementary Table S2), and a clustering heatmap was then drawn based on the relative classification results of the first 20 ranked wax components. As depicted in Figure 4B, the relative content of the first 20 compounds were all decreased after infection with *B. cinerea* for 15 and 30 d. Figure 4C–E shows the relative content of fatty acids, esters, and olefins during the experiment. Although there were no significant differences between the two groups on the content of fatty acids at 15 d, the content of fatty acids decreased significantly at 30d in the *B. cinerea*-inoculated group, and the relative content of esters and olefins significantly decreased at 15 and 30 d.

### 2.5. Differential Gene Expression Analysis of B. cinerea-Inoculated ‘Munage’ Grapes

**Figure 5:**
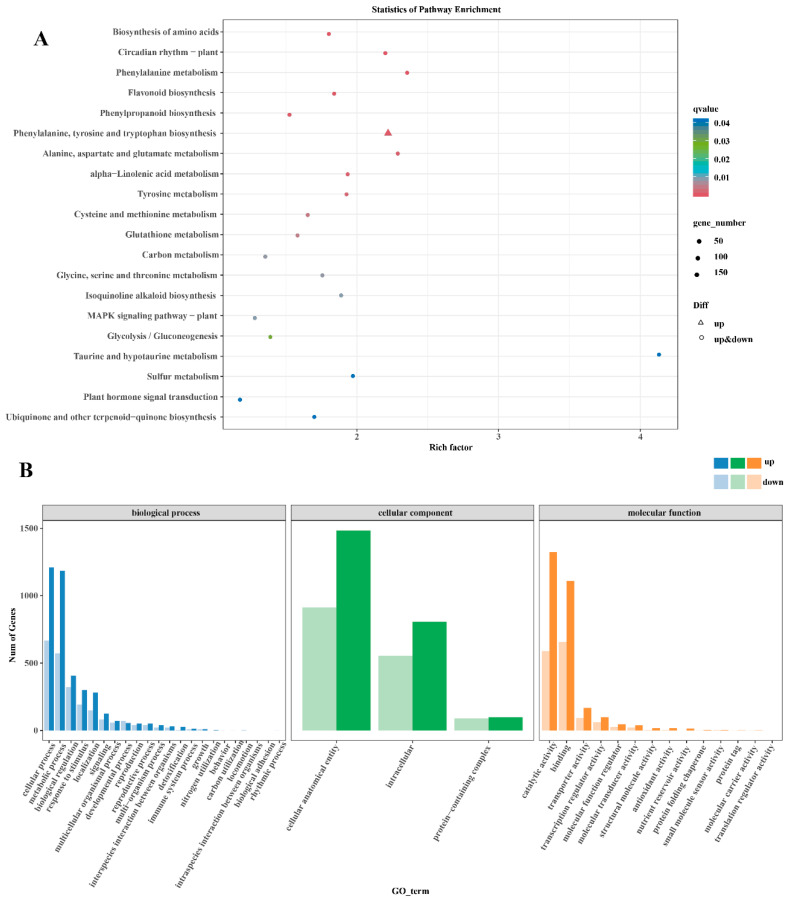
Kyoto Encyclopedia of Genes and Genomes (KEGG) and Gene Ontology (GO) in CK15_VS_BC15. (**A**) Enrichment of KEGG. (**B**) Enrichment of GO.

To further explore the regulation mechanism of *B. cinerea* molecules on grape epidermal wax, RNA-seq was performed on the tested grapes, and the differential gene analysis of *B. cinerea*-infected grapes is shown in Figure 5. Principal component analysis (PCA) revealed distinct clustering of the sample distribution in CK and BC groups at 15 d (Supplementary Figure S1A). As demonstrated by the volcano plot and heatmap depicted in Supplementary Figure S1B, compared with the CK, 4334 DEGs were detected in the *B. cinerea*-inoculate group, of which 2699 genes were upregulated and 1635 genes were downregulated.

The 431 DEGs analyzed by KEGG enrichment are shown in Figure 5A. According to the KEGG metabolic pathway and differential gene expression analysis, this study found 24 key DEGs involved in the synthesis of 13 and extension of 11 fatty acids from the CK15_vs_BC15 group (Table 1). The genes of long-chain acyl-CoA synthase (*LACS*), 3-ketoacyl-CoA synthase (*KCS*), and acetyl-CoA carboxylase (*ACC*) were mainly enriched in the KEGG pathway of fatty acid synthesis, in which *KCS20*, *LACS6*, and *ACC1* were up-regulated, while ECR and LACS7 were down-regulated. Additionally, 14 DEGs were involved in the synthesis of cuticle and wax, mainly the ultra-long chain aldehyde decarbonylase (*CER3*), alkane hydroxylase (*MAH1*), O-acyltransferase (*WSD1*), and cytopigment (*CYP94A1*). *MAH1* and *WSD1* were down-regulated, while *CER3* and *CYP94A1* were up-regulated. Furthermore, 11 DEGs were involved in wax transport, mainly non-specific lipid transfer proteins (*LTPG1*, *LTP1*) and the G subfamily of ABC transporters (*ABCG*). *LTPG1* was down-regulated, *LTP1* was up-regulated, and most *ABCGs* were up-regulated.

Gene Ontology (GO) functional annotation technology was used to classify the overall functions of DEGs, and it was divided into biological processes (BPs), cellular components (CCs), and molecular functions (MFs). Dominant functional areas were further subdivided into multiple subcategories. For the DEGs in this group, 17 BPs, 3 CCs, and 14 MFs were enriched. The BPs of “cellular processes” and “metabolic processes” were enriched; the CCs of “cellular anatomical entity” and “intracellular” were highly enriched; and the MFs of “catalytic activity” and “binding” were significantly enriched (Figure 5B).

### 2.6. Differential Transcription Factors Analysis of B. cinerea-Inoculated ‘Munage’ Grapes

The expression levels of transcription factors (TFs) in CK15_VS_BC15 fruit samples were analyzed, and a total of 12 different TF families with significant differences were identified by comparison and screening (Table 2). Among these differentially expressed transcription factors, the *bHLH*, *ERF*, *WRKY*, *TCP*, and *MYB* families showed outstanding enrichment effects. In addition, this study found that most transcription factors, such as *bHLH41*, *ERF98*, and *WRKY24*, were significantly up-regulated in this group, while *MYB308* and *TCP4* were significantly down-regulated. It is speculated that these genes play an important role in responding to pathogen invasion and regulating wax synthesis.

### 2.7. Validation of DEGs and TFs

**Figure 6:**
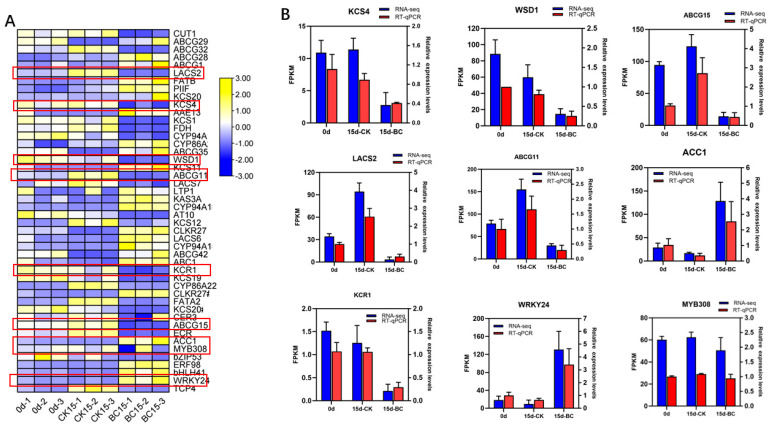
qRT-PCR validation of DEGs and TFs. (**A**) The expression levels of DEGs and TFs in CK15_VS_BC15. (**B**) qRT-PCR was used to verify nine genes involved in fatty acid elongation, synthesis of wax, and transcription factors.

To confirm the reproducibility and accuracy of the RNA-seq analysis results, nine genes implicated in fatty acid elongation, synthesis of wax, and transcription factors were chosen for subsequent quantitative (qRT-PCR) assays (Figure 6A). Simultaneously, the Fragments Per Kilobase of exon per Million fragments mapped (FPKM) values from the RNA-seq results were compared with those of the qRT-PCR results to ascertain whether the gene and TF expression patterns corresponded to the sequencing results (Figure 6B). The decreasing expression trends of *KCS4*, *WSD1*, *ABCG15*, *LACS2*, *ABCG11*, *KCR1*, and *MYB308* derived by both RNA-Seq and qRT-PCR after *B. cinerea* infection were consistent, and the expression of *ACC1* and *WRKY24* increased following the same trend.

### 2.8. The Potential Mechanism of B. cinerea-Induced Wax Metabolism in ‘Munage’ Grapes

**Figure 7:**
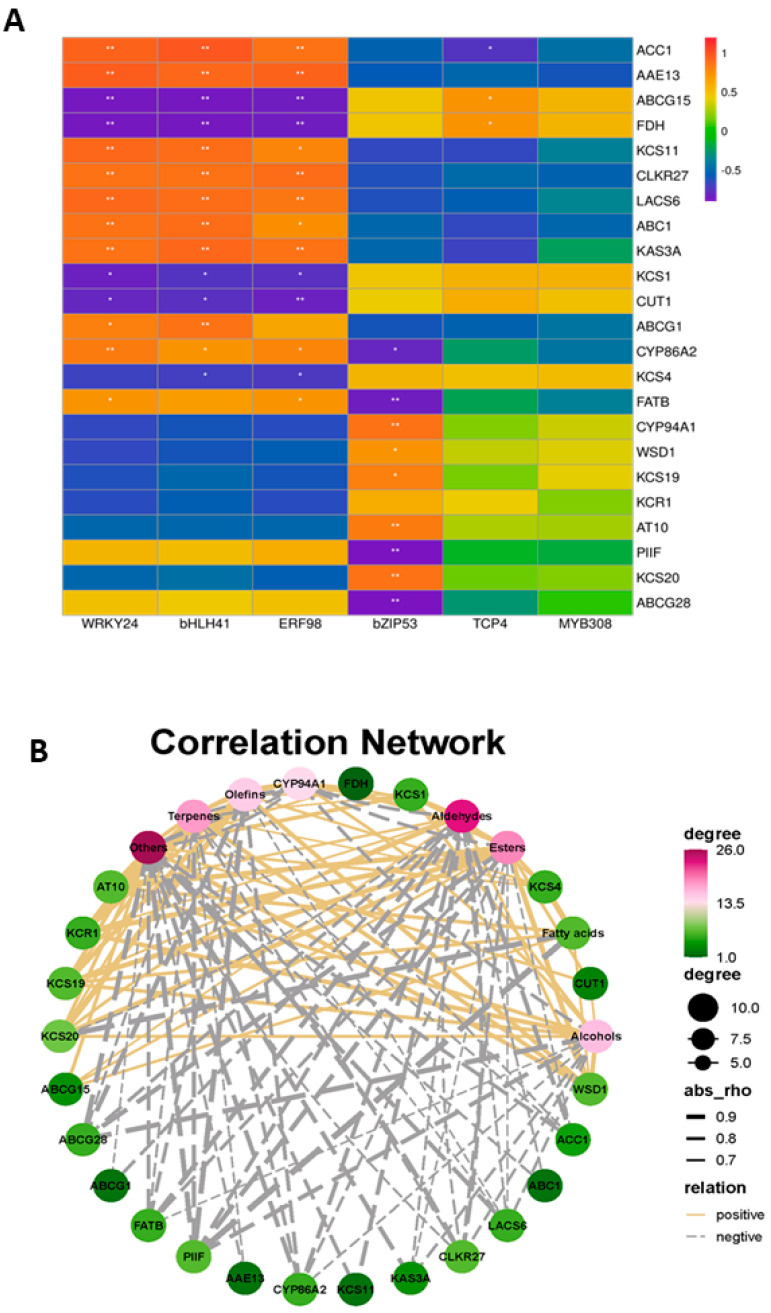
Correlation analysis between TFs, waxy components, and waxy-related DEGs. (**A**) The relationship between TFs and waxy-related DEGs. (**B**) The relationship between waxy components and waxy-related DEGs. * and ** denote significance at *p* < 0.05, *p* < 0.01.

**Figure 8:**
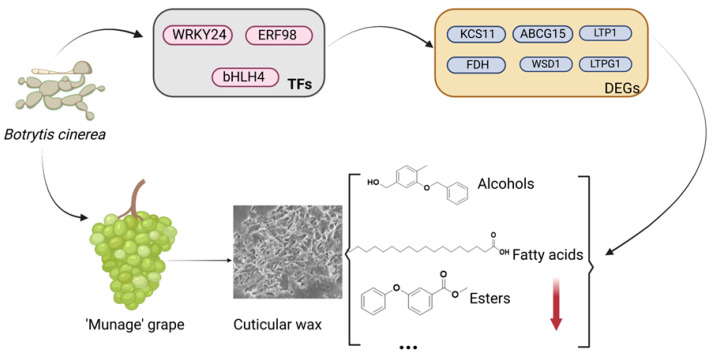
The potential mechanism of *B. cinerea*-induced wax metabolism in ‘Munage’ grapes.

As shown in Figure 7A, the gene relating to fatty acid elongation, FDH, was negatively correlated with *WRKY24*, *bHLH4*, and *ERF98*, while *KCS11* was positively correlated with *WRKY24*, *bHLH4*, and *ERF98*. The genes relating to fatty acid synthesis, *CLKR27*, *LACS6*, *AAE13*, and *ACC1*, had a strong positive relationship with *WRKY24*, *bHLH4*, and *ERF98*. To clarify, the transcription factors played a vital role in *B. cinerea*-inoculated grapes, and we further explored the relationship between transcription factors and genes related to wax synthesis. The associations between the wax components and genes associated with wax transport altered by *B. cinerea* were determined by PLS correlation analysis (Figure 7B). The abundance of aldehydes, esters, alcohols, terpenes, and other wax components, including fatty acids and olefins, had high correlations with the genes involved in wax synthesis. The potential molecular mechanisms underlying *B. cinerea*-induced wax metabolic reprogramming in ‘Munage’ grapes are illustrated in Figure 8. The schematic diagram depicts a regulatory cascade where *B. cinerea* infection triggers differential expression of TFs, including *WRKY24*, *bHLH4*, and *ERF98*. These TFs subsequently modulate key wax biosynthesis-related genes, including *KCS11*, *ABCG15*, *LTP1*, *FDH*, *WSD1*, and *LTPG1*. This transcriptional regulation ultimately disrupts the wax biosynthetic pathway and changes the composition of the ‘Munage’ grapes’ epicuticular wax.

## 3. Discussion

‘Munage’ grapes are a kind of grape variety with local characteristics favored by consumers in recent years because of its sweet taste. However, table grapes are susceptible to fungi and pathogenic bacterial infection during post-harvest storage, which may affect their quality and selling price [5]. The infection of *B. cinerea* in grapes during post-harvest storage is considered to be the key factor responsible for fungi infection, but the exact mechanism of *B. cinerea* infecting ‘Munage’ grapes is still unclear [18]. In this study, after inoculation with *B. cinerea* for 15 d, the weight loss rate and color difference significantly increased in ‘Munage’ grapes, while the firmness significantly decreased compared with grapes in the CK group. The decrease in fruit firmness during *B. cinerea* infection involves both structural degradation and targeted biochemical manipulation. For instance, *B. cinerea* suppresses the activity of pectin methylesterase inhibitor (PGIP), an enzyme critical for blocking cell wall degradation. In infected fruits, cellulose and total pectin content significantly decline, while polygalacturonase (PG) activity rises sharply [2]. The synergistic effect of these biochemical attacks and *B. cinerea* penetration degrade the fruit epidermal tissue, thus accelerating the decline of fruit firmness. Furthermore, compared with the control group, H_2_O_2_ and O_2_^−^ of ‘Munage’ grapes in the inoculation group were significantly increased. The reactive oxygen species (ROS) burst may be mediated by NADPH oxidase activation in the plasma membrane, which serves as a key signal transduction node. Concomitantly, the antioxidant enzymes SOD, GSH, CAT, and POD showed biphasic changes (initial activation followed by down-regulation), representing a sophisticated adaptive strategy integrating both protective scavenging and signal propagation functions during pathogen challenge.

Cuticular wax serves as the initial obstacle against pathogen invasion into fruit and has been verified as one of the significant elements influencing pathogen resistance [19]. Besides antifungal properties of cuticular wax, the composition and quantity of wax were greatly changed after fungi infection. The wax structure of ‘Munage’ grapes in this study was flaky and stratified by many tubules. After 15 d of continuous infection of *B. cinerea*, we found the epidermal wax was “melting” and surrounded by mycelium. Furthermore, the epidermal wax dissolved and disappeared from *B. cinerea*-infected grapes; these results suggest that during cold storage, *B. cinerea* spores germinated on the surface of the wax to form mycelium, which might further secrete the cutinase to dissolve wax, and therefore with the storage time, the epidermal wax appears to melt until the phenomenon of serious disappearance. A recent study found that ‘Hongshuijing’ pitaya with thicker cuticular wax layers and higher wax content were less susceptible to decay than ‘Baishuijing’ pitaya [20]. Our results showed that the wax concentration significantly decreased in *B. cinerea*-infected ‘Munage’ grapes after 15 and 30 d of cold storage, suggesting that wax accumulation and cutin layer thickness differences caused by table fruit variety and storage conditions may affect the susceptibility of pathogenic bacteria. It is well known that the epicuticular wax is important for maintaining the quality of grapes after harvest, which is mainly composed of fatty acids [21]. Our study revealed that *B. cinerea* infection alters the composition of ‘Munage’ grapes’ epicuticular wax—a critical barrier against pathogen invasion [22]. Relevant studies have associated *B. cinerea* with reduced wax components and postharvest quality decline [22,23]. In our study, *B. cinerea* significantly decreased the relative contents of fatty acids on day 30, while the relative contents of esters and olefins significantly decreased on days 15 and 30. These results suggest that *B. cinerea* may degrade epicuticular wax components to compromise defense mechanisms of ‘Munage’ grapes, thereby accelerating disease progression and decay development during cold storage.

Given that epicuticular wax plays a crucial role in fruit quality, it is of great significance to understand the molecular mechanism by which *B. cinerea* induces wax loss in ‘Munage’ grapes. According to the omics profiles, we found the *B. cinerea* regulated 4334 DEGs of ‘Munage’ grapes, including up-regulating 2699 genes and down-regulating 1635 genes. KEGG showed significant enrichment of plant hormone signal transduction and interaction with plant bacteria. In addition, the KEGG pathway enrichment analysis also involved the MAPK signaling pathway, biological synthesis of amino acids, and biosynthesis of phenylpropane in plants. Fatty acid metabolism had been shown to induce the skin waxes in postharvest apples [22]. VLCFA biosynthesis in epicuticular wax has been widely reported, as *KCS* and *ECR* are involved in chain elongation, and *LACS* and *ACC* are involved in fatty acid synthesis [23]. In this study, the genes *KCS20*, *LACS6*, and *ACC1* were up-regulated, while *ECR* and *LACS7* were down-regulated, indicating that *B. cinerea* affected the synthesis and extension of fatty acids of ‘Munage’ grapes during cold storage after harvest. Cuticle waxes are the outermost substances involved in plant–pathogen interactions. Greater wax coverage leads to a thicker lipid barrier layer, which helps slow the quick breakdown of the cuticle; we found the genes related to the synthesis of cuticle and wax, including *MAH1*, *WSD1, CER3*, and *CYP94A1*, were up-regulated or down-regulated after infection with *B. cinerea*. In particular, the expression of *WSD1* was reduced in the *B. cinerea* group, which is consistent with previously reported changes in gene expression in wax metabolism after fungal infection [24]. The G family of ATP-binding cassette proteins (ABCG transporters) on plant cell membranes can transfer wax and cutin precursors from the membrane. Herein, our results showed that *B. cinerea* infection increased the expression of *LTP1* and most *ABCGs*, implying the wax components of ‘Munage’ grapes are conveyed to the exterior of epidermal cells by means of ABCG transporters and lipid transfer proteins (LTPs). It has been well accepted that the WRKY protein represents one of the most extensive plant-specific transcription factor groups and holds crucial functions in plant stress reactions. Additionally, the WRKY family has been discovered to be actively involved in modulating plant basal resistance against *B. cinerea* by interacting with differentially expressed genes (DEGs) associated with wax synthesis [25]. In our study, the RNA-Seq data of *WRKY24* were significantly up-regulated in the *B. cinerea* infection group, suggesting that *WRKY24* plays an important role in regulating resistance caused by *B. cinerea* in ‘Munage’ grapes. It is noteworthy that other TFs, including *bHLH41* and *ERF98*, were also increased, while *MYB308* and *TCP4* were decreased. In order to clarify the accuracy of RNA-seq, we randomly selected DEGs, including *KCS4*, *WSD1, ABCG15, LACS2, ABCG11, KCR1*, and *MYB308*, and TFs, including *ACC1* and *WRKY24*, for validation by qRT-PCR. As expected, the relative gene expression levels aligned with the overall trend outcomes of the transcriptome. Moreover, qRT-PCR analysis validated that the gene expression profiles derived from the transcriptome were reliable and convincing.

To further investigate the relationship between DEGs, TFs, and epidermal wax components, our test is based on exploring the correlation of *B. cinerea*-influenced TFs via up-regulating or down-regulating DEGs with the changes in epidermal wax components like fatty acids, esters, and olefins after *B. cinerea* infection. As shown in Figure 7, *WRKY24*, *bHLH4*, and *ERF98* were positively correlated with *KCS11* and negatively correlated with fatty acid elongation *FDH*, indicating that *WRKY24*, *bHLH4*, and *ERF98* play an important role in response to pathogen invasion and regulation of wax synthesis. Furthermore, the DEGs involved had a positive or negative correlation with the contents of wax components, including aldehydes, esters, alcohols, terpenes, fatty acids, and olefins. This study revealed the mechanism of *B. cinerea* infection in ‘Munage’ grapes and provided countermeasures for reducing the adverse effects of fungal infection on grape quality and improving post-harvest preservation technology, which could further improve the economic benefits of the grape industry.

## 4. Materials and Methods

### 4.1. Grapes and Chemical Substances

‘Munage’ grapes were purchased from Jinma market (Shihezi, China). For the present study, grapes that were consistent in color, free from pests and diseases, and without any mechanical damage were selected and stored in cold storage at 4 °C. *B. cinerea* (provided by Grape and Wine Research Center at the School of Food Science and Technology, Shihezi University) was cultured for 7 days and prepared in a spore suspension (1 × 10^6^ CFU mL^−1^). All grapes underwent standardized surface sterilization with 75% ethanol to eliminate pre-existing microbial communities. For the BC treatment group, the grapes’ surfaces were evenly sprayed with *B. cinerea* suspension, while the control group (CK group) was treated with an equivalent volume of sterile water. The microstructure and content of wax in grapes’ epidermis were observed at 0, 15, and 30 days.

### 4.2. Determination of Postharvest Quality in ‘Munage’ Grapes

The disease occurrence rate was evaluated as the proportion (%) of the quantity of fruit exhibiting pathogen growth on the surface in relation to the overall fruit quantity. The outcome was appraised as the percentage (%) of the fruit amount with pathogen development on the surface with respect to the total fruit amount [16]. The rate of fruit deterioration during storage was evaluated based on the area of decay (primarily resulting from infection) on the fruit exterior. The fruit decay rate was ascertained using the following grading system: 1, surface without any decay; 2, 0–25% decay; 3, 25–50% decay; 4, 50–75% decay; and 5, >75% decay. The fruit decay index was computed as: DI = (decay score × corresponding number of fruit within each category)/(total number of fruit × scale values) × 100 [26]. The loss in fruit weight was determined through the weighing approach. The result was noted as the percentage (%) of the weight after storage in comparison to the weight before storage. Grapes were haphazardly chosen from each treatment cohort, and the firmness was measured by the GY-4 hardness tester (Yueqing Aidibao Instrument, Wenzhou, China) [27,28]. The color index was calculated with ΔE = (ΔL)^2^ + (Δa)^2^ + (Δb)^2^, where “L” denotes the luminosity, “a” indicates the red-green shade, and “b” represents the yellow-blue tint [28]. Each grape was compressed over a distance of 5 mm at the equator with a measuring speed of 1.0 mm s^−1^, and the firmness was represented as the maximum force (N) during the probe’s compression process [29].

### 4.3. Analysis of Defense Enzyme Activities

The H_2_O_2_ content (BC3595) was determined using a hydrogen peroxide assay kit (Sorabio Technology, Beijing, China) The absorbance at 405 nm was used to calculate the sample content (μmol/kg) based on the standard. For O_2_^−^ determination, the original method was adjusted. First, 5.0 g grape samples were weighed, mixed with 5.0 mL extraction buffer for grinding, and the centrifuged supernatant was reserved. Then, 1.0 mL of the supernatant was mixed with 1.0 mL of 50 mmol/L, pH 7.8 phosphoric acid buffer, then 1.0 mL of 1 mmol/L hydroxylamine hydrochloride solution was added, and the mixture was left at 25 °C for 1 h. After that, 1.0 mL of 17 mmol/L p-aminobenzenesulfonic acid and 1.0 mL of 7 mmol/L alpha-naphthylamine were added, mixed well, and left at 25 °C for 20 min for color reaction. The absorbance of the color-developing solution at 530 nm was measured using a standard curve-like preparation method, with results expressed in nmol/min·g.

For SOD activity determination, 5.0 g grape samples were mixed with 5 mL of 0.1 M sodium phosphate buffer (pH 7.8), centrifuged, and the supernatant was used for enzyme activity measurement. Then, 50 mM phosphate buffer (pH 7.8), 130 mM methionine, 750 μM NBT, 100 μM EDTA-Na_2_, and 20 μM riboflavin were added to the supernatant. Three tubes for determination and two for control were placed under a 4000—l× fluorescent lamp for 15 min and then in the dark to end the reaction. For GSH content determination, 5.0 g fruit samples were weighed into a mortar, ground with 5.0 mL of 5% (*w*/*v*) trichloroacetic acid solution (with 5 mM EDTANa_2_) precooled at 4 °C, and centrifuged, and the supernatant was collected for GSH detection. The GSH content in fruit tissue (μmol/g) was calculated based on the absorbance difference and the standard curve. CAT activity was measured using a catalase kit (Sorabio Technology, Beijing, China). For POD activity determination, the extraction buffer was made of 1 mmol PEG 6000, 4% PVPP, 1% Triton X-100, and 0.1 M sodium acetate-acetic acid buffer (pH 5.5). Then, 5.0 g grape samples were mixed with 5.0 mL extraction solution and centrifuged, and the supernatant was used for the enzyme activity assay. After adding 5 mL enzyme extract, 3.0 mL guaiacol (25 mM), and 200 μL H_2_O_2_ (0.5 M), the reaction was started.

### 4.4. Determination of Wax Microstructure and Total Content

The microstructure of the ‘Munage’ grapes’ surface was investigated via scanning electron microscopy (SEM). With blade tweezers, grape samples were meticulously excised into 3 × 3 mm pieces, and the excess fluid on the tissue was carefully blotted using paper towels [30]. The peel sections were prepared at three time points: 0 d, which corresponded to the harvest stage, and 15 and 30 d after inoculation with *B. cinerea*. Subsequently, the samples were sputter-coated with a gold–palladium alloy (60:40) using a LEICA EM ACE600L coater (Wetziar, Germany). Finally, the surface microstructures were visualized and analyzed using a field emission SEM system (JEOL JSM-6360LV, Tokyo, Japan) at an accelerating voltage of 10 kV.

Dichloromethane (Beilian Fine Chemical Development, Tianjin, China) was mixed with n-hexane (Beilian Fine Chemical Development, Tianjin, China) in a volume ratio of 3:1 as a solvent for extracting wax using a liquid–solid ratio of 2:1 (volume/weight). The grape granules were placed into a beaker with a capacity of 500 mL, and the solvent prepared in advance was slowly added [24,31]. The extraction time was 7.5 min. After soaking, the grapes were carefully removed with tweezers to avoid breakage. The wax extracts were filtered by quantitative filter paper and stored at 4 °C without oxygen until further analysis.

### 4.5. Analysis of Wax Components in ‘Munage’ Grapes

A gas chromatograph-mass spectrometer (QP2020NX, SHIMADZU, Kyoto, Japan) outfitted with an Rtx-5 MS capillary column was employed to analyze the wax samples [7]. The inlet temperature was held steady at 250 °C. A constant flow rate of 1 mL min^−1^ was set for the helium carrier gas. Concerning the temperature program of the gas chromatograph, it commenced at 70 °C and remained so for 1 min. Then, it was increased at a rate of 10 °C per minute until reaching 200 °C. Following that, the temperature was escalated at a rate of 4 °C per minute until it hit 300 °C and was maintained at this level for 20 min. The mass spectrometer was set to operate in the positive electron ionization mode with the parameters set to EI (70 eV, *m*/*z* 45–650). The temperature of the transfer line was retained at 280 °C, and the ion source was held at 200 °C [32].

To identify the wax compounds, their mass spectra were compared with those in the NIST17 MS library. For the evaluation of retention indices of the detected wax compounds, a homologous series of n-alkanes was used as an internal standard.

### 4.6. RNA Sequencing (RNA-Seq) Analysis

Total RNA was extracted from grape samples using a Trizol kit (Vazyme Biotech, Nanjing, China) followed by quality assessment of RNA purity and integrity. mRNA enrichment was performed using Oligo(dT)-coated magnetic beads, then fragmented chemically. First-strand cDNA synthesis was conducted with reverse transcriptase, followed by second-strand synthesis using DNA polymerase I. After magnetic bead purification, the double-stranded cDNA underwent end repair (T4 DNA polymerase), A-tailing (Klenow polymerase), and Illumina adapter ligation. Fragments (200–300 bp) were selected using AMPure XP beads, followed by PCR amplification with Phusion High-Fidelity DNA polymerase to construct the final library [33,34]. The data analysis was conducted using the BMKCloud platform (www.biocloud.net). Cleaned sequencing data were aligned to the reference genome (Vitis_vinifera.IGGP_12x.23.genome.fa) for subsequent analyses, including library quality evaluation, structural characterization, differential expression analysis, and functional enrichment using established pipelines. Then, the RSEM tool was used to quantify the expression of these genes, the expression level of genes was measured by FPKM, and the genes with significant differences in expression levels were identified and analyzed (DEGs). A gene was defined as a DEG only if FDR ≤ 0.05 and |log2(FC)| ≥ 1. Finally, GO (Gene Ontology) and KEGG (Kyoto Encyclopedia of Genes and Genomes) enrichment analyses were performed for these DEGs using the GOseq R v4.0.4. and KOBAS 3.0 tool [35].

### 4.7. qRT-PCR Validation

To confirm the dependability of the RNA-seq data, seven differentially expressed genes (DEGs) and two transcription factors (TFs) were arbitrarily chosen from the outcomes of the transcription study for qRT-PCR verification. The specific primers for the DGEs and Pg Actin (the internal control gene) are presented in Supplementary Table S1. Primer Premier 5.0 primer design software was employed to formulate primers for the selected genes with differential expression. GAPDH was adopted as the internal reference gene, and the relative expression level was computed using the 2^−∆∆CT^ approach [36].

### 4.8. Statistical Analysis and Correlation Analysis

The data were presented in the format of means ± standard deviations (SDs). For the determination of significant differences among groups, Fisher’s Least Significant Difference (LSD) test was implemented via SPSS 19.0. A p value lower than 0.05 was deemed statistically significant. In order to screen the potential mechanism of *B. cinerea* infection on the wax of ‘Munage’ grapes, we used the data of BC15 and CK15 to assess the relationship between DEGs, TFs, and wax components. A partial least-squares regression correlation analysis and correlation network were carried out using R v4.0.4.

## 5. Conclusions

This work revealed the components of *B. cinerea*-infected wax on the surface of ‘Munage’ grapes during storage at 4 °C. In conclusion, infection with *B. cinerea* decreased the disease incidence, decay rate, and amount of wax in ‘Munage’ grapes. *B. cinerea* also changed the principal wax components of ‘Munage’ grapes, as revealed by the significant decreases in fatty acids, esters, and olefins were in the BC group. Additionally, the TFs, including WRKY24, bHLH4, and ERF98, might play a significant role in regulating genes related to wax synthesis and altering the wax composition. Overall, this study investigated the underlying resistance mechanisms of transcriptional changes in ‘Munage’ grapes to *B. cinerea* infection and provides new insights into potential resistance mechanisms.

## Figures and Tables

**Table 1:** DEGs related to fatty acid synthesis extension, wax synthesis, and transport in CK15_VS_BC15.

Types of Participation	Gene ID	Gene	Log_2_FC	Annotated Gene Function
Fatty acid elongation	VIT_18s0001g07640	*KCR1*	−1.37	Very-long-chain 3-oxoacyl-CoA reductase
	VIT_04s0008g02250	*FDH*	−3.36	3-ketoacyl-CoA synthase
	VIT_07s0141g00060	*KCS20*	7.32	3-ketoacyl-CoA synthase
	VIT_07s0141g00090	*KCS20*	−4.12	3-ketoacyl-CoA synthase
	VIT_11s0016g04700	*KCS11*	2.42	3-ketoacyl-CoA synthase
	VIT_13s0019g01260	*ECR*	−1.14	Very-long-chain enoyl-CoA reductase
	VIT_13s0067g03890	*KCS19*	−3.08	3-ketoacyl-CoA synthase
	VIT_14s0006g02990	*CUT1*	−2.29	3-ketoacyl-CoA synthase
	VIT_15s0048g02720	*KCS1*	−1.86	3-ketoacyl-CoA synthase
	VIT_18s0001g12550	*KCS4*	−1.64	3-ketoacyl-CoA synthase
	VIT_06s0004g04000	*KCS12*	−2.97	3-ketoacyl-CoA synthase
Fatty acid synthesis	VIT_00s0233g00060	*KAS3A*	1.16	3-oxoacyl-[acyl-carrier-protein] synthase
	VIT_00s2731g00010	*FATA2*	−1.21	Oleoyl-acyl carrier protein thioesterase
	VIT_03s0063g01880	*LACS2*	−4.39	Long chain acyl-CoA synthetase
	VIT_05s0020g03080	*LACS6*	1.02	Long chain acyl-CoA synthetase
	VIT_08s0007g07520	*At3g03980*	4.51	NADPH-dependent aldehyde reductase-like protein
	VIT_08s0007g07530	*At3g03980*	1.05	NADPH-dependent aldehyde reductase-like protein
	VIT_08s0040g01190	*CLKR27*	1.65	3-oxoacyl-[acyl-carrier-protein] reductase
	VIT_08s0040g01200	*CLKR27*	3.67	3-oxoacyl-[acyl-carrier-protein] reductase
	VIT_09s0002g04170	*AAE13*	1.82	Malonate-CoA ligase
	VIT_11s0016g04480	*FATB*	1.31	Palmitoyl-acyl carrier protein thioesterase
	VIT_14s0128g00720	*LACS7*	−1.03	Long chain acyl-CoA synthetase
	VIT_17s0000g01090	*FATB*	−8.09	Palmitoyl-acyl carrier protein thioesterase
	VIT_18s0001g04980	*ACC1*	3.47	Acetyl-CoA carboxylase
Synthesis of cuticle and wax	VIT_11s0037g01210	*CER3*	3.14	Very-long-chain aldehyde decarbonylase
	NewGene_1726	*MAH1*	−1.02	Alkane hydroxylase
	VIT_00s0207g00010	*AT10*	−3.22	Acyl transferase
	VIT_02s0025g03320	*CYP86A22*	−4.41	Cytochrome
	VIT_05s0020g05040	*PIIF*	1.67	Wound-induced proteinase inhibitor
	VIT_06s0009g03630	*CYP94A1*	−1.25	Cytochrome
	VIT_07s0141g00890	*CYP94A1*	6.62	Cytochrome
	VIT_07s0141g00920	*CYP94A1*	4.52	Cytochrome
	VIT_15s0046g02380	*CYP86A2*	1.27	Cytochrome
	VIT_15s0046g00480	*WSD1*	−1.59	O-acyltransferase
	VIT_15s0046g00520	*WSD1*	−6.13	O-acyltransferase
	VIT_15s0046g00590	*WSD1*	−8.08	O-acyltransferase
Lipid transport	NewGene_222	*LTPG1*	−1.70	Non-specific lipid-transfer protein
	VIT_14s0108g00520	*LTP1*	1.74	Non-specific lipid-transfer protein
	VIT_06s0061g00230	*ABCG15*	−2.65	ABC transporter G family member
	VIT_09s0002g03550	*ABCG35*	3.10	ABC transporter G family member
	VIT_09s0002g03560	*ABCG42*	2.99	ABC transporter G family member
	VIT_09s0002g03570	*ABCG29*	3.06	ABC transporter G family member
	VIT_11s0016g04540	*ABCG32*	−1.44	ABC transporter G family member
	VIT_13s0019g04600	*ABCG28*	1.14	ABC transporter G family member
	VIT_13s0067g03750	*ABCG1*	3.67	ABC transporter G family member
	VIT_16s0039g00010	*ABCG11*	−1.85	ABC transporter G family member
	VIT_07s0005g02430	*ABC1*	1.01	Protein ABC transporter

**Table 2:** TFs related to grapes stress resistance and wax regulation.

TFs ID	TFs	Log_2_FC	Annotated TF Function
VIT_03s0038g02310	*MYB308*	−1.02	transcription factor
VIT_11s0016g02070	*bHLH41*	4.96	transcription factor
VIT_05s0049g00500	*ERF98*	6.83	ethylene-responsive transcription factor
VIT_08s0058g00690	*WRKY24*	4.31	transcription factor
VIT_19s0014g01680	*TCP4*	−2.16	transcription factor
VIT_03s0038g04450	*bZIP53*	1.85	bZIP transcription factor

## Data Availability

The data used to support the findings of this study can be made available by the corresponding author upon request.

## Supplementary Materials

The following supporting information can be downloaded at: https://www.mdpi.com/article/10.3390/ijms26083468/s1.
