# Supplementary material for: Post-Harvest Quality Changes and Molecular Responses of Epidermal Wax in ‘Munage’ Grapes with Botrytis cinerea Infection
Source: Int J Mol Sci. 2025 Apr 8;26(8):3468. doi: 10.3390/ijms26083468 (PMC12026965; doi:10.3390/ijms26083468)
Supplement: Supplementary file 1 [file ijms-26-03468-s001.zip › Supplementary Figure.pdf]

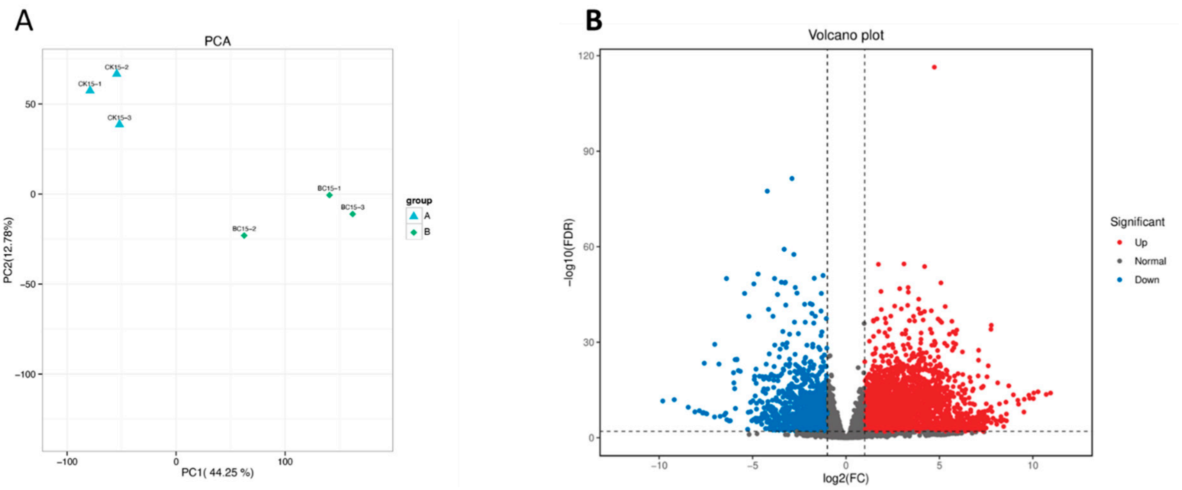

Supplementary Figure S1. Principal component analysis (PCA) and volcano plot in CK15_VS_BC15. (A) PCA, (B) volcano plot.
