# Supplementary material for: Post-Harvest Quality Changes and Molecular Responses of Epidermal Wax in ‘Munage’ Grapes with Botrytis cinerea Infection
Source: Int J Mol Sci. 2025 Apr 8;26(8):3468. doi: 10.3390/ijms26083468 (PMC12026965; doi:10.3390/ijms26083468)
Supplement: Supplementary file 1 [file ijms-26-03468-s001.zip › Supplementary Table.pdf]

Supplementary Table S1 Primer sequences used for qRT-PCR

Gene	Forward Primer (5’-3’)	Reverse Primer (5’-3’)
KCS4	TGCTGGAGGAAGGGCTGTTA	TGCCAGACACGATTACCCTT
WSD1	TTGCTCCAAGTTGCTATGGG	GAGGGTCAGGAATGGTCGC
MYB308	ATATCTTTCGCAGCCGCAGT	GGTTGGTATGGTGGGCTGAT
ABCG11	AAGCCTGTGTGGCGTTATCC	TTGGGAAGACCATTCGGTGT
ABCG15	CTGTCACTCTTTTTTTCTGTG	TACCACATCAGTCGTCTTC
LACS2	GTACCTTCACCACTCCTGGC	GCAGGCCCAGTAGTTCAGTT
ACC1	ATGGGCGGAATGGTCTCTTTC	TGGGGACCTTGTCTTCATCAT
KCR1	CGGAGAAGAACCACTTCGAG	AAGCATCCCTCCTCCTTGAT
WRKY24	CTCCGCAGAAGATTGAAGAG	TGTGTGGGGCAAAATGGCT
Actin	TCCTTGCCTTGCGTCATCTAT	CACCAATCACTCTCCTGCTACAA

Supplementary Table S2 Effects of *Botrytis cinerea* infection on grape wax compounds

Number	Waxy compound name	Molecular formula	0d	CK-15d	BC-15d	CK-30d	BC-30d
Classification: Fatty acid							
1	Lauric acid	$C_{12}H_{24}O_2$	2.64±0.43	2.32±0.31	1.34±0.20	1.13±0.15	0.95±0.13
2	Tetradecanoic acid	$C_{14}H_{28}O_2$	4.72±0.84c	2.57±0.43b	2.78±0.37b	2.24±0.23b	0.78±0.21a
3	Palmitic acid	$C_{16}H_{32}O_2$	22.58±1.69c	14.37±1.06b	15.48±1.31b	11.58±1.23b	7.66±0.56a
4	Octadecanoic acid	$C_{18}H_{36}O_2$	19.90±1.37c	14.05±1.25b	14.56±1.64b	10.73±1.20b	5.92±0.24a
5	Arachidic acid	$C_{20}H_{40}O_2$	10.99±1.99c	6.36±1.23b	6.83±1.30b	5.41±0.87b	2.90±0.34a
6	Behenic acid	$C_{22}H_{44}O_2$	12.08±2.18b	9.11±1.55b	9.28±1.61b	6.27±1.23a	5.56±1.04a
7	Tetracosanoic acid	$C_{24}H_{48}O_2$	10.53±1.73b	6.81±1.22a	7.02±1.34a	5.89±0.95a	2.38±0.40a
8	Hexacosanic acid	$C_{26}H_{52}O_2$	10.58±1.83b	7.03±1.06a	7.24±1.35a	5.65±0.45a	3.23±0.32a
9	Montanic acid	$C_{28}H_{56}O_2$	11.90±2.11b	7.94±1.23a	8.80±1.41a	6.21±1.01a	4.74±0.33a
10	Myricyl acid	$C_{30}H_{20}O_2$	13.38±2.05c	8.49±1.42b	8.64±1.20b	7.32±0.78bb	4.96±0.36a
11	Oleic acid	$C_{18}H_{34}O_2$	9.08±1.64	6.31±1.23	6.05±0.35	5.37±0.56	3.02±0.21
12	Linoleic acid	$C_{18}H_{32}O_2$	8.10±1.32	6.34±1.34	6.46±0.46	4.31±0.33	3.78±0.22
13	Palmitoleic acid	$C_{16}H_{30}O_2$	1.29±0.12	1.09±0.44	1.13±0.19	0.78±0.46	0.67±0.10
14	Erucic acid	$C_{22}H_{42}O_2$	4.18±0.95	1.48±0.36	ND	ND	ND
Classification: Olefins							
15	n-Tetradecane	$C_{14}H_{30}$	1.07±0.18	0.78±0.24	0.56±0.45	0.54±0.31	0.16±0.05
16	n-Hexadecane	$C_{16}H_{34}$	1.82±0.31	1.47±0.26	0.67±0.32	0.80±0.24	0.47±0.13
17	n-Heptadecane	$C_{17}H_{36}$	7.09±0.78b	4.81±0.61a	3.35±0.34a	3.94±0.52a	1.64±0.20a
18	n-Nonadecane	$C_{19}H_{40}$	7.18±0.90	5.38±0.70	3.97±0.32	3.63±0.23	2.87±0.12
19	4, 6-Dimethyldodecane	$C_{14}H_{30}$	1.36±0.12	1.01±0.16	0.87±0.20	0.57±0.11	0.29±0.03
20	2,6, 11-Trimethyldodecane	$C_{15}H_{32}$	2.54±0.13	1.27±0.14	1.05±0.10	1.08±0.07	0.55±0.03

Number	Waxy compound name	Molecular formula	0d	CK-15d	BC-15d	CK-30d	BC-30d
21	2,6,10,14Tetramethylheptadecane	$C_{21}H_{44}$	3.10±0.63	2.78±0.15	2.93±0.32	1.34±0.21	0.93±0.08
22	2,3,5, 8-Tetramethyldecane	$C_{13}H_{28}$	2.35±0.54	1.97±0.16	1.56±0.14	1.34±0.15	0.51±0.21
23	Cyclododecane	$C_{12}H_{24}$	1.37±0.20	0.95±0.54	1.02±0.32	0.74±0.14	0.65±0.12
Classification: Alcohols							
24	2-Methyltetradecan-1-ol	$C_{15}H_{32}O$	1.28±0.17	1.08±0.23	0.78±0.10	0.69±0.32	0.48±0.04
25	3, 5-Dimethyl-3-hexanol	$C_8H_{18}O$	1.83±0.32	1.02±0.35	0.95±0.26	0.56±0.37	0.31±0.11
26	2, 2-Dimethyl octanol	$C_{10}H_{22}O$	7.37±1.28b	6.68±1.05b	4.22±0.78b	4.02±0.69b	1.96±0.40a
27	cis-11-Tetradecene-1-ol	$C_{14}H_{28}O$	2.14±0.40	2.04±0.37	1.90±0.17	1.16±0.12	0.65±0.30
28	Eicosanol	$C_{20}H_{42}O$	1.01±0.26	0.78±0.24	0.95±0.43	0.34±0.05	0.31±0.02
29	Cis-9-tetradecenol	$C_{14}H_{28}O$	2.16±0.34	1.86±0.22	1.87±0.26	0.86±0.07	0.65±0.06
30	Phytol	$C_{20}H_{40}O$	0.55±0.10	ND	ND	0.16±0.03	ND
31	2-Methyl-1-decanol	$C_{11}H_{24}O$	1.27±0.25	1.02±0.45	0.78±0.32	0.57±0.20	0.24±0.31
32	2-Hexyl-1-octanol	$C_{14}H_{30}O$	5.35±1.06	4.21±0.54	2.37±0.34	2.70±0.46	1.98±0.20
Classification: Esters							
33	Methyl octacoate	$C_{29}H_{58}O_2$	0.35±0.10	0.27±0.04	0.26±0.06	0.15±0.01	0.13±0.03
34	Eicosyl acetate	$C_{22}H_{44}O_2$	3.81±0.72	3.05±0.31	3.24±0.30	1.84±0.34	1.23±0.15
35	Dioctyl phthalate	$C_{24}H_{38}O_4$	2.74±0.38	2.31±0.20	2.57±0.41	1.78±0.10	1.06±0.06
36	Methyl 12-methyltridecanoate	$C_{15}H_{30}O_2$	3.45±0.63b	3.21±0.19b	2.46±0.11b	2.01±0.09b	0.76±0.14a
37	Dibutyl phthalate	$C_{16}H_{22}O_4$	16.35±1.20c	12.31±1.04c	6.76±0.16b	5.32±0.32b	1.99±0.12a
38	Methyl 3-(3, 5-di-tert-butyl-4-hydroxyphenyl) propionate	$C_{18}H_{28}O_3$	15.30±1.01b	12.82±1.32b	8.64±0.60b	5.35±0.34a	1.87±0.12a
39	Methyl oleate	$C_{19}H_{36}O_2$	6.74±0.67b	5.29±0.46b	3.75±0.37a	1.01±0.08a	0.85±0.05a
Classification:Aldehydes							
40	Hendecanal	$C_{17}H_{34}O$	0.73±0.02	0.54±0.05	0.47±0.02	0.32±0.11	0.18±0.03

Number	Waxy compound name	Molecular formula	0d	CK-15d	BC-15d	CK-30d	BC-30d
41	Nonal dimethyl acetal	$C_{11}H_{24}O_2$	3.36±0.63	3.19±0.31	2.79±0.24	1.57±0.21	1.31±0.20
42	Trans-2-nonylaldehyde	$C_9H_{16}O$	0.84±0.12	0.73±0.20	0.54±0.15	0.38±0.36	0.26±0.02
43	Pentadecanal	$C_{15}H_{30}O$	15.63±1.43b	13.51±1.25b	7.30±0.56a	6.52±0.34a	3.61±0.14a
44	13-Methyltetradecral	$C_{15}H_{30}O$	0.78±0.10	0.67±0.12	0.39±0.03	0.32±0.04	ND
Classification: Terpenes							
45	Isolongifolene	$C_{15}H_{24}$	6.57±1.37b	5.62±0.92a	5.21±1.49a	3.15±0.31a	1.24±0.07a
46	Isopinocampheol	$C_{10}H_{18}O$	0.86±0.14	0.56±0.11	0.45±0.13	0.35±0.15	ND
47	Alpha, 2, 5-Trimethylphenylpropenone	$C_{12}H_{14}O$	10.34±1.95c	8.04±1.02c	4.56±0.56b	5.42±0.31b	1.04±0.50a
Classification: Others							
48	1, 2-Octadecane oxide	$C_{18}H_{36}O$	15.76±1.38b	13.30±1.49b	6.19±0.62a	5.73±0.66a	1.41±0.27a
49	2, 6-di-tert-Butylp-cresol	$C_{15}H_{24}O$	1.72±0.30	1.64±0.31	1.35±0.55	0.96±0.80	0.22±0.04
50	Testosterone Ketolaurate	$C_{12}H_{24}O$	0.33±0.12	0.30±0.07	0.24±0.06	0.12±0.09	ND
51	Methyl arachidate	$C_{20}H_{40}O$	0.34±0.09	0.29±0.04	0.17±0.12	0.13±0.13	ND
52	1, 1-Dimethoxysolane	$C_{12}H_{26}O_2$	1.57±0.31	1.35±0.21	ND	0.65±0.11	ND
